# Evaluating Health Literacy among Adolescent and Young Adult Pregnant Women from a Low-Income Area of Northeast Brazil

**DOI:** 10.3390/ijerph17238806

**Published:** 2020-11-27

**Authors:** Allen Suzane França, Catherine M. Pirkle, Tetine Sentell, Maria P. Velez, Marlos R. Domingues, Diego G. Bassani, Saionara M. A. Câmara

**Affiliations:** 1 Postgraduate Program in Public Health, Faculty of Health Sciences of Trairi, Federal University of Rio Grande do Norte, Santa Cruz, Rio Grande do Norte CEP 59200-000, Brazil; allensuzanefranca@gmail.com; 2 Office of Public Health Studies, University of Hawaii at Manoa, Honolulu, HI 96822-2319, USA; cmpirkle@hawaii.edu (C.M.P.); tsentell@hawaii.edu (T.S.); 3 Department of Obstetrics and Gynaecology, Queen’s University, Kingston General Hospital, Kingston, ON K7L 2V7, Canada; maria.velez@queensu.ca; 4 Department of Public Health Sciences, Queen’s University, Kingston General Hospital, Kingston, ON K7L 2V7, Canada; 5 Postgraduate Programme in Physical Education, Federal University of Pelotas, Pelotas, Rio Grande do Sul CEP 96055-630, Brazil; marlosufpel@gmail.com; 6 Centre for Global Child Health, The Hospital for Sick Children, Toronto, ON M5G 0A4, Canada; diego.bassani@sickkids.ca; 7 Department of Paediatrics, Faculty of Medicine & Dalla Lana, School of Public Health, University of Toronto, Toronto, ON M5G 0A4, Canada

**Keywords:** health literacy, adolescent pregnancy, health disparities, social determinants of health

## Abstract

Adequate health literacy is important for strong health outcomes during pregnancy, particularly among mothers with high risk of adverse outcomes related to pregnancy and childbirth. Understanding the health literacy of young pregnant women in low-income settings could support strategies to reduce adverse outcomes in this population. This exploratory study assessed the health literacy of young pregnant adolescents and young adults from a rural area in Northeast Brazil and associated factors such as socioeconomic conditions, adequacy of prenatal care, and social support from family and friends. In this cross-sectional study, 41 pregnant adolescents (13–18 years) and 45 pregnant adults (23–28 years) from the Rio Grande do Norte state, Brazil, were assessed regarding health literacy through the Short Assessment of Health Literacy for Portuguese-Speaking Adults (SAHLPA, score from 0–18, inadequate if <15). Income sufficiency, self-perceived school performance, compliance with recommendations for adequate prenatal care, and social support were also assessed. A linear regression analysis was conducted to evaluate the variables associated with the SAHLPA score. Ninety-five percent of the adolescents and 53.3% of the adults (*p* < 0.001) presented inadequate health literacy. Adolescent age (β − 3.5, *p* < 0.001), poorer self-perceived school performance (β − 2.8, *p* < 0.001), and insufficient income for basic needs (β − 2.8, *p* = 0.014) were associated with worse SAHLPA scores. Adolescent mothers have higher rates of inadequate health literacy in this population. Policies are needed to improve access to health information for young populations from rural low-income areas.

## 1. Introduction

The World Health Organization defines “health literacy” as “the personal characteristics and social resources needed for individuals and communities to access, understand, appraise, and use information and services to make decisions about health” [1]. Thus, on the one hand, individuals with adequate health literacy levels are better equipped to manage their health and the health of their families and communities [2]. On the other hand, low health literacy is associated with lower use of health services [3,4], increased medical costs [5], and low self-rated general health [6], as well as social disadvantage (such as low income and education levels), where disadvantage is known to contribute to poorer health outcomes at all ages [7].

Excellent health literacy requires being able to read, write, fill out forms, and comprehend health information, all necessary skills to understand health-related materials and to act efficiently in different situations [8]. The ability to interpret, filter, judge, and evaluate health information is also critical [8]. Adolescent and young adult years are critical for the development of social, emotional, and cognitive skills that are necessary to have autonomy over health and to establishing healthy patterns of behavior over the life-course [9,10]. Adequate health literacy during these years can help reduce environmental and interpersonal barriers that young people often face when interacting with health systems [9]. As recognized by others [8,9], little is known about health literacy during adolescence and this knowledge gap is particularly acute in low- and middle-income countries. Moreover, there is a particular need for research that examines predictors of health literacy in adolescence [9]; identification of adolescent-specific predictors of health literacy requires comparison with other age groups. 

Among young populations, low sexual and reproductive health literacy is associated with unintended pregnancies which are, in turn, associated with adverse social and health outcomes for the mother and the child [11]. Worldwide, 11% of all pregnancies are among adolescents aged 15–19 years, and about 95% of these pregnancies occur in low- and middle-income countries [12]. The higher rates of adolescent fertility among less advantaged populations may indicate, among other issues, a lack of knowledge about contraception, lack of access to care, or an inability to navigate health services, as well as limited knowledge of the effects of pregnancy and childbirth at younger ages on health-related outcomes [13,14,15,16].

Despite some studies [11,17] that have investigated the level of sexual and reproductive health literacy among pregnant adolescents, to date, as far as we know, there has not been a study that has assessed the level of general health literacy in this population. Higher health literacy is associated with positive health behaviors in adolescents, including lower use of harmful and addictive substances [18]. Thus, it is likely that higher levels of health literacy among pregnant adolescents are associated with more positive health behaviors during pregnancy and may help to reduce the adverse health-related outcomes associated with pregnancy and childbirth among this population. The aim of this study was to assess the health literacy of young pregnant adolescents (ages 13–18 years) and a comparable group of young pregnant adults (ages 23–28 years) from a rural area in Northeast Brazil and to examine associated factors, such as socioeconomic conditions, adequacy of prenatal care, and social support from family and friends. 

## 2. Materials and Methods

### 2.1. Study Site and Design

This is an exploratory cross-sectional analysis of the Adolescent and Motherhood Research (AMOR) project, a pilot prospective cohort study that was designed to test the feasibility of research that could be used to test the hypothesis that adolescent pregnancy increases the risk of chronic conditions and mobility loss over the life-course. The work presented here is secondary data analysis from this cohort study.

The study was performed in the Trairi region of the Rio Grande do Norte state, located in Northeast Brazil, which is a rural low-income area. Data were collected in five small cities of this region, whose populations varied from 4500 to 40,000 inhabitants for each city [19].

The AMOR participants were recruited up to the 16th week of pregnancy with two additional follow-up visits, i.e., after 27 weeks of pregnancy (during the third trimester) and between 4 and 6 weeks postpartum. Data on health literacy were collected in the second round of data collection (third trimester of pregnancy) when cumulative exposure to health information via prenatal care providers was expected to be highest. Because only a single wave of data was collected on health literacy in this pilot study, this is a cross-sectional analysis of the data. 

### 2.2. Population and Sample

The study population at baseline (<16 weeks pregnant) was composed of 100 pregnant adolescents and young adults living in the five cities of the region. This sample size was considered to be sufficiently powered to assess the validity of epidemiological instruments [20], which was one of the main objectives of the AMOR project. Fifty primigravid adolescents (ages 13 to 18 years), and 50 pregnant adults (ages 23 to 28 years) attending antenatal care within the primary health care system of these municipalities were recruited following referral from the primary health care providers or self-referral in response to a study advertisement displayed at local health care units and community centers. The age range for the adolescent group was based on previous research showing that childbirth-related risks decline dramatically at and after the age 18 [21]. For the adult group, the age range was defined based on evidence suggesting this age group was at lowest childbirth-associated risk of chronic disease [22,23,24], which was one of the main objectives of the AMOR project. While this study takes advantage of the AMOR data to examine the health literacy data collected during the third trimester of pregnancy, the design of the AMOR study is particularly relevant to the research objective of this study, that is, the comparison group of young adult pregnant women from the same rural region in Brazil allows for inferences to be drawn on adolescent specific correlates of health literacy. 

Participants were considered ineligible for the pilot if they had a diagnosis of psychiatric (including prolonged use of antidepressant and anxiolytic medications) or chronic conditions (hypertension, diabetes, HIV/AIDS, heart disease, tuberculosis, cancer, epilepsy, and lupus) before pregnancy, during their first prenatal visit. 

**Figure 1:**
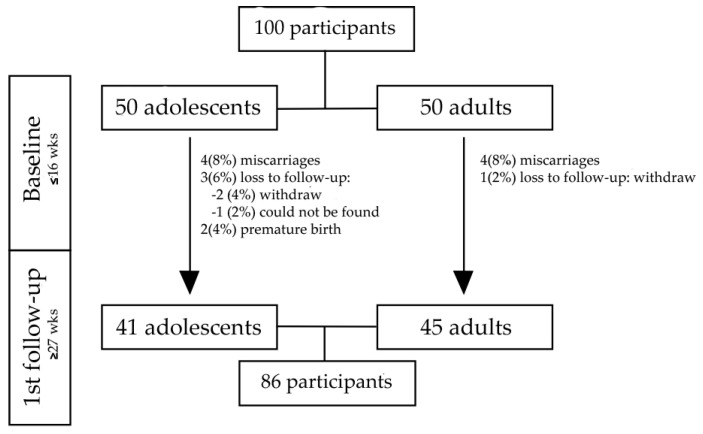
Flowchart of sample by age group within the Adolescent and Motherhood Research (AMOR) study in the Trairi region, Rio Grande do Norte, Brazil, 2017–2018.

From the 100 eligible participants that consented and were included in the baseline, eight were excluded because they had a miscarriage before the second evaluation, three for withdrawing from the study, and one could not be found for follow-up assessment. Another two participants had a premature birth before the last trimester of pregnancy, and therefore were not evaluated at this stage of pregnancy. Thus, the sample of the present study was composed of 86 pregnant women, in their third trimester, i.e., 41 adolescents and 45 adults (Figure 1).

### 2.3. Procedures

Before data collection, the interviewers were trained on the research protocols and standardized procedures including following ethical protocols for enrollment of human subjects in research studies [25]. Participants were interviewed at the primary health care units of the municipalities or at the Faculty of Health Sciences of Trairi, a campus of the Federal University of Rio Grande do Norte located at Santa Cruz. Participants unable to reach one of these locations, due to transportation or health challenges, were interviewed in their homes (*n* = 17). Data collection followed the standardized protocol described below.

#### 2.3.1. Socioeconomic Data

Socioeconomic data were collected through a structured questionnaire programmed into Qualtrics software [26], and included the following variables: age and race/skin color, which were self-reported. Self-perceived school performance as compared with peers was assessed through the question, “How do you evaluate your performance at school compared to your classmates?” Responses were categorized as “better than average” and ”average/worse”. Income sufficiency was assessed using the question, “To what extent does your financial situation meet your needs?” The possible responses were the following: very well, adequately, not very well. and not at all, further grouped as very well, suitable and not well for the analysis. Benefits received from the ”Bolsa Família” Program were collected by asking participants if someone in their household was currently receiving Bolsa Família (yes or no). The ”Bolsa Família” program is a conditional cash transfer program, created in 2003, with the objective of fighting poverty and reducing inequalities by providing cash payments to poor families (up to 89 Brazilian Reals per person per month, equivalent to 14.9 Euros or 16.8 US Dollars by July 2020) [27].

#### 2.3.2. Prenatal Care Adequacy

This was evaluated by verifying how the procedures and exams performed during pregnancy adhered to the national recommendations for adequate prenatal care [28]. For this, information from the pregnant women’s prenatal card was collected after the delivery and the record of the presence of the following procedures was collected: attendance at least six prenatal consultations during pregnancy, attendance at three prenatal education meetings, one blood type (ABO) and Rh factor laboratory assessment, one urine bacterial culture test, one cervical-vaginal cytopathology, one obstetric ultrasound, one hepatitis B surface antigen test (HBsAg), one serology for toxoplasmosis (IgM), two urine tests, two blood glucose levels, two venereal disease research laboratory (VDRL) tests, two hemoglobin/hematocrit, and two anti-human immunodeficiency virus (HIV). We assessed the record of the compliance with each of the 13 recommendations as recorded in the participants prenatal cards and created a variable describing the proportion of all recommendations followed and registered in the prenatal cards. Thus, this variable ranged from 0, when no recommendations were registered in the prenatal card, to 100, when all 13 recommendations were registered. 

We also recorded the number of prenatal consultations performed during pregnancy and included it as a continuous variable in the analysis.

#### 2.3.3. Social Support

Because peers and family are known to influence health literacy [9], social support was assessed via a short version of the Social Networks and Social Support tool used in the International Mobility in Aging Study [29], which evaluates social networks and social support provided by different types of social ties (friends, family, and partner). For the present study, only social support was assessed, asking each participants if she feels loved and appreciated by her friends, family, and partner; if they listen to the her when she needs to talk about her problems or concerns; if she helps her friends, family and partner; if she feels that she plays an important role in their lives; and if she feels useful to them. For each question, five answers were possible, i.e., never, rarely, sometimes, often, and always, with a score ranging from 0 (never) to 4 (always). The final score was converted into quartiles for each type of relation and the quartile values were dichotomized as high social support (quartiles 2, 3, and 4) and low or no social support (quartile 1).

#### 2.3.4. Health Literacy

Health literacy was evaluated using the Short Assessment of Health Literacy for Portuguese-Speaking Adults (SAHLPA-18) [30], which has been validated for the Brazilian population. In this validation work, the SAHLPA had a high correlation with formal education (Spearman’s r = 0.62) and self-reported functional literacy (Spearman’s r = 0.74). The SAHLPA-18 also presented good internal consistency (Cronbach’s alpha = 0.90) and test-retest reliability (intraclass correlation coefficient = 0.91, 95% CI 0.76, 0.96), with the cutoff of ≤14 presenting 83.3% sensitivity and 66.7% specificity to identify inadequate health literacy. The SAHLPA-18 evaluates pronunciation and comprehension skills of 18 common medical terms. The test was performed from printed cards, each with a medical term printed in bold at the top and two words of association at the bottom. Participants were asked to pronounce the word in bold, and then the interviewer pronounced the two words of association and asked the participant to say which of the two words was more related to the word in bold. For example, the interviewer shows the card with the word OSTEOPOROSIS. The participant should pronounce that word correctly, and then say if it is related to BONE or MUSCLE, which were the two options of association words written on the bottom. The answer was considered to be correct when the medical term was pronounced correctly, and the associated word was correct. The terms included in the SAHLPA-18 are osteoporosis, pap smear, miscarriage, hemorrhoids, abnormal, menstrual, behavior, seizure, rectal, appendix, arthritis, caffeine, colitis, gallbladder, jaundice, prostate, incest, and testicle. Each correct item received 1 point and the total score was obtained by summing all items, ranging from 0 to 18 points. Following previous work, participants who scored from 0 to 14 points were classified as having inadequate health literacy and those who scored between 15 and 18 points were classified as having adequate health literacy [30].

### 2.4. Ethics

All participants were informed about the objectives and procedures of the survey on the first contact. The study protocol was approved by the Ethics and Research Committee of the Faculty of Health Sciences of Trairi at the Federal University of Rio Grande do Norte and by the National Council of Ethics in Research, with approval number 2.628.406. All participants were informed about the study procedures and those who agreed to participate signed the study consent form (adult participants) or the assent form (adolescent participants). All legal representatives of the adolescent participants also signed a consent form, following the Brazilian ethical rules.

### 2.5. Data Analysis

Data were computed and analyzed using the Statistical Package for Social Sciences (SPSS version 25.0, IBM Corporation, New York, NY, USA). First, descriptive statistics for all variables were estimated for each age group (adolescents and adults). We estimated medians and 25th and 75th percentiles for continuous variables and absolute and relative frequency for categorical variables. The normality of data distribution was assessed using the Kolmogorov–Smirnov test. To evaluate the association between health literacy and covariates, we used the Mann–Whitney test for the continuous variables and the Chi-square test for the categorical ones. The medians of the dependent variable (SAHLPA-18 score) were presented according to the categories of the independent variables and compared using the Mann–Whitney or Kruskal–Wallis tests. Multiple linear regression was performed to evaluate the association between the independent variables and the SAHLPA-18 score. All variables associated with health literacy in the bivariate analysis were included in the initial model and those that were non-significant were eliminated by the backward stepwise method. Only those that showed statistical significance with the SAHLPA-18 score remained in the final model. A significance level of *p* < 0.05 was set for all statistical analyses.

## 3. Results

Sample characteristics are presented in Table 1. Compared to pregnant adolescents, adult pregnant women were more likely to have higher compliance with the national recommendations for adequate prenatal care (61.54% vs. 38.46%), and greater social support from parents (82.2% vs. 61.0%) and friends (75.6% vs. 48.8%). No significant differences were observed between adults and adolescents for race/color, self-perceived school performance as compared with peers, income sufficiency, receiving Bolsa Família, number of prenatal consultations, and social support from grandparents, partner or siblings.

Seventy-three percent of the sample (N = 63) had inadequate health literacy. A significantly higher proportion of adolescents (N = 39, 95.1%) presented inadequate health literacy as compared with adults (N = 24, 53.3%). Table 2 presents the association among the independent variables and health literacy. The proportion of pregnant women who reported average/low school performance as compared with peers, received Bolsa Família, and reported low/no social support of parents was significantly higher among participants with inadequate health literacy. No statistically significant differences were found among health literacy groups in relation to race, income sufficiency, number of prenatal consultations, and social support from friends, grandparents, partners and siblings.

The median SAHLPA-18 score was 10.5 (range 7–15). Adult pregnant women had higher median scores than adolescents (Table 2). Similarly, pregnant women with self-perceived school performance better than average obtained a higher score than those reporting school performance as average/low. The median SAHLPA-18 score was significantly higher among pregnant women who reported having suitable income as compared with those that reported having either a very high or very low income. The median score among participants reporting not receiving Bolsa Família was significantly higher than those reporting receiving it. There was no significant difference in median score by type of social support. However, a marginally higher median score was observed for pregnant women who had high social support of parents as compared with those with low parental support (*p* = 0.052) Appendix A shows the comparisons of SAHLPA scores between adolescents and adults for all categories of the independent variables (Table A1). Significantly lower SAHLPA scores were observed for adolescents in almost all categories, showing that pregnant adolescents presented poorer health literacy than pregnant adults in most socioeconomic subgroups.

Women with adequate health literacy also presented higher compliance with recommendations for an adequate prenatal care than those classified as having inadequate health literacy (65.38 (53.85–76.92 vs. 46.15 (30.77–69.23), *p* = 0.014) (data not shown in the table).

Table 3 shows the multiple linear regression results for the SAHLPA-18 score. In the final model, age group, self-perceived school performance, and income sufficiency remained significantly associated with SAHLPA-18 score. Adolescent participants scored, on average, 3.5 points lower on the SAHLPA-18 than adults, even after adjustment for covariates. Perceiving school-performance better than average was associated with higher health literacy, with a SAHLPA-18 score almost 3 points higher than for those who perceived their school performance average/low. Moreover, the SAHLPA-18 scores of pregnant women who reported suitable income were almost 3 points higher as compared with those reporting insufficient income.

## 4. Discussion

This study investigated health literacy and its associated factors in pregnant adolescents and adults living in a rural low-income area in Northeastern Brazil. The results revealed low levels of health literacy among this population, with adolescent participants presenting worse results than adults. Almost all of the adolescent respondents (95.1%) had low health literacy. Lower health literacy was also associated with worse self-perception of school performance as compared with peers, receiving Bolsa Família, having lower social support from parents, and having had a lower record of recommendations for an adequate prenatal care in the pregnant cards in the bivariate analysis. In the multiple linear regression model, age group, self-perception school performance, and income sufficiency remained associated with health literacy. 

When comparing our results with previous studies [31,32,33,34,35] that evaluated health literacy in adolescents and adults, we observed that our sample presented a higher prevalence of inadequate health literacy. We found that 73.3% of our sample had inadequate health literacy, including 95.1% of adolescents and 53.3% of adults. Studies performed with pregnant adults [36,37,38,39] reported inadequate health literacy ranging from 14% to 61% using different instruments, such as the Rapid Estimate of Adult Literacy in Medicine (REALM) [40] and the Test of Functional Health Literacy in Adults (TOFHLA) [41]. The REALM evaluates the ability to pronounce some medical words. This is different from the SAHLPA-18 used in the present study, which evaluates both, pronunciation and comprehension of medical terms. The TOFHLA incorporates a different concept to evaluate health literacy, i.e., assessing the person’s ability to read and comprehend some medical instructions such as those for treatment and exams. As far as we know, there are no studies evaluating health literacy among pregnant women with SAHLPA-18. Although there are validated versions of the TOFHLA [42] and SAHLPA for the Brazilian population, it has been reported that the TOFHLA may be intimidating to people with lower education and it may have limited application for some vulnerable populations in developing countries [30], such as those evaluated in the present study. Thus, it is possible that even higher percentages of low health literacy would have been found if we have evaluated the participants of the present study with the Brazilian version of the TOFHLA. 

Regarding health literacy among adolescents, previous studies [23,34,43,44,45] found rates of low health literacy varying from 23% to 48% in males and females. Although the high prevalence of inadequate health literacy in our sample may reflect poorer results for rural low-income participants, they may also reflect differences in relation to the health literacy tests used by the previous studies, which, in some cases, may use simpler questions and commands. For instance, when using the REALM-TEEN [43], a previous study with 293 adolescents (14–19 years) from the United States, the authors reported a prevalence of health literacy of 24.2%. This instrument is a version of the REALM adapted to teenagers and evaluates only the ability to pronounce words. However, other studies with more complex health literacy tests, such as the Newest Vital Sign [23], which tests literacy skills for numbers and words, and C-sTOFHLAd [44], which assesses reading comprehension in two reading passages related to medical instructions, also found lower levels of health literacy in adolescents than seen in this study.

Our adolescent group had lower health literacy than the adults in the sample. Other studies have also found an association between age and health literacy. A previous study of adolescents aged 15 to 19 years found that poorer health literacy was associated with younger ages [45], as did another study with adolescents aged 11 to 18 years [46]. This may reflect the lower access to health information at younger ages, as well as developmental immaturity and less experience/interaction with the health system. 

Poorer results among the younger participants could reflect the association between lower education level and health literacy. Education and health literacy are different concepts; therefore, the isolated analysis of education level does not necessarily explain health literacy [47]. Health literacy develops from the intersection of several essential components related to the broad concept of literacy such as cultural and conceptual knowledge, listening, speaking, reading, writing and numeracy skills [48]. Therefore, greater access to education could expose people to more health information. We did not include years of schooling in this analysis because of the collinearity with the participants’ age. This is also concerning as these are pregnant adolescents and having a baby may impact their academic trajectories.

It is well stablished that adolescent mothers already face greater risks related to pregnancy and childbirth, as well as their children. The incidence of adverse health conditions such as eclampsia and systemic infections are higher among adolescent mothers as compared with adults [49]. Preterm birth, stillbirths, and newborn deaths are more frequent among adolescents as well [50,51]. As lower health literacy is associated with less healthy choices, riskier health behavior and more inappropriate use of health services [52], therefore, low health literacy may increase the risks associated with adolescent fertility for the mother and the child. Improving health literacy in the young pregnant population is an important intervention that might improve their health outcomes as well as that of their child. Health literacy is a factor that can be intervened in policy and practice before, during, and after adolescent pregnancy for potential positive health impacts immediately and over time. The low health literacy of this population must also be recognized, and health care systems must use this information to consider health interventions for improving health literacy, and also to ensure that health materials and health systems are easy-to-use and understandable for individuals across all health literacy skills. Health professionals, services, organizations, and systems must make health information and resources available and accessible to people, according to their health literacy strengths and limitations.

Regarding the perception of school performance, the present study revealed poorer health literacy among those reporting average/low school performance as compared with peers. As pointed out in previous studies [53,54,55], negative perceptions can compromise students’ self-esteem, behavior, and motivation for learning. With this, the negative self-perception of school performance may also have discouraged the search for health information, and thus led to poorer health literacy. 

The analyses indicate that a lower SAHLPA-18 score is associated with a lower income sufficiency and receiving Bolsa Família, which agrees with existing literature [31,56]. The association between health literacy and income sufficiency remained after multivariate linear regression analysis. This association is explained by people with lower income having less opportunity to access health information and services or less support for health-related decision making, which consequently reduces the chances of developing health literacy. Socioeconomic inequality requires targeted interventions that address the specificities of people with inadequate health literacy.

In the bivariate results, health literacy was lower in pregnant women who also had a poor record of compliance with prenatal recommendations, i.e., worse adequacy of prenatal care. Women with limited health literacy may have less knowledge about some screening tests in the first and second trimesters of pregnancy [57]. Another study indicated that pregnant women with low health literacy started prenatal care later than pregnant women with adequate health literacy [58]. Thus, it is possible that pregnant women with lower health literacy were less concerned about carrying out all the tests and procedures recommended for an adequate prenatal care. The lack of association, in the multivariate analysis, may indicate the collinearity between adequacy of prenatal care and the age group. As presented in Table 1, the adequacy of prenatal care was significantly worse for adolescents than for adults.

As for social support, pregnant women with inadequate health literacy had significantly lower social support from parents, although the association lost significance in the adjusted model. Other studies with non-pregnant populations have found a direct relationship between health literacy and social support [59,60,61]. Lack of support from family may hinder the process of acquiring health-related information.

This study presents some limitations. Because the SAHLPA-18 instrument was developed to measure health literacy among adults, the poorer results found for adolescents may be overestimated. However, the SAHLPA is composed of simple medical terms normally used in routine health care consultations and procedures, and the inability of pregnant adolescents to understand these terms reflects an important health concern. This instrument does not evaluate other critical components and skills of health literacy, such as numeracy, language skills, and research skills, and we believe that looking at other aspects of health literacy in this population is an important topic for the future. Finally, the small sample size is another limitation of this study, which limited the power of our analyses and also limits the generalization or the results.

## 5. Conclusions

We found lower health literacy among pregnant adolescents as compared with adults, with a greater proportion of the adolescent sample having low health literacy. Poorer results were also associated with self-perceived school performance as compared with peers equal to others or worse and worse perception of income sufficiency. These results indicate the need for policies that target improvement of health information access, engagement, and understanding for young populations from rural low-income areas, where the rates of adolescent pregnancy are particularly high. Strategies must respond to locally identified health literacy needs and focus on increasing equity in health outcomes and access to services for people with varying health literacy levels. Actions must be planned to make information and services more available and accessible and to enhance the ability and willingness of young populations to engage with health-related information and services available, to communicate and assert their health decisions, and to take appropriate actions to implement the decisions they make about their health [1]. With this, specific programs for young mothers, including age-appropriated approaches and information provided during prenatal consultations, must be implemented among prenatal services. Consistently using plain language and universal precautions, especially given the high levels of low health literacy across groups, would be helpful to address the issues identified in this article, as would building health care organizations that are responsive to diverse client needs and preferences.

## Figures and Tables

**Table 1:** Sample characteristics according to age groups, Trairi region, Rio Grande do Norte, Brazil, 2017–2018 (N = 86).

Characteristics	Total	Adolescents (N = 41)	Adults (N = 45)	
	N (%) or Median (q_25_:q_75_) ^a^			*p* Value
Age	23 (17:25)	17 (16:18)	25 (24:26)	<0.001 ^e^
Race/color ^b^				
White	29 (34.1)	11 (26.8)	18 (40.9)	0.171 ^c^
Mixed-race/Black	56 (65.9)	30 (73.2)	26 (59.1)	
Self-perceived school performance compared to peers				
Better than average	39 (45.3)	15 (36.6)	24 (53.3)	0.119 ^c^
Average/worse	47 (54.7)	26 (63.4)	21 (46.7)	
Income sufficiency				
Very well	19 (22.1)	7 (17.1)	12 (26.7)	0.101 ^c^
Suitable	55 (64.0)	25 (61.0)	30 (66.7)	
Not well	12 (14.0)	9 (22.0)	3 (6.7)	
Receiving “Bolsa Família” ^b^				
Yes	56 (65.9)	31 (75.6)	25 (56.8)	0.068 ^c^
No	29 (34.1)	10 (24.4)	19 (43.2)	
Number of antenatal consultations ^b^				
≥6	33 (38.8)	17 (41.5)	16 (36.4)	0.630 ^c^
<6	52 (61.2)	24 (58.5)	28 (63.6)	
Proportion of adequacy of antenatal care ^d^	53.84 (38.46:69.23)	38.46 (23.08:69.23)	61.54 (46.15:69.23)	0.005 ^e^
Social Support				
Friends				
High	54 (62.8)	20 (48.8)	34 (75.6)	0.010 ^c^
Low/none	32 (37.2)	21 (51.2)	11 (24.4)	
Grandparent				
High	57 (66.3)	31 (75.6)	26 (57.8)	0.081 ^c^
Low/none	29 (33.7)	10 (24.4)	19 (42.2)	
Parents				
High	62 (72.1)	25 (61.0)	37 (82.2)	0.028 ^c^
Low/none	24 (27.9)	16 (39.0)	8 (17.8)	
Partner				
High	59 (68.6)	26 (63.4)	33 (73.3)	0.322 ^c^
Low/none	27 (31.4)	15 (36.6)	12 (26.7)	
Siblings				
High	61 (70.9)	26 (63.4)	35 (77.8)	0.143 ^c^
Low/none	25 (29.1)	15 (36.6)	10 (22.2)	
Total	86 (100)	41 (47.7)	45 (52.3)	

^a^ q25, q75: 25th and 75th percentiles; ^b^ one missing data; ^c^ Chi-square test; ^d^ seven missing data; ^e^ Mann–Whitney test.

**Table 2:** Associations among health literacy and independent variables (N = 86).

Characteristics	Health Literacy					
	Adequate N = 23	Inadequate N = 63	*p* Value	SAHLPA-18 Score ^a^ (0–18)		*p* Value
	**N (%)**			**Median**	**q_25–_q_75_**	
Age categories						
Adults	21 (46.7)	24 (53.3)	<0.001 ^b^	13	09–16	<0.001 ^c^
Adolescents	2 (4.9)	39 (95.1)		08	06–11	
Race/color ^d^						
White	6 (20.7)	23 (79.3)	0.432 ^b^	10	08–13	0.524 ^c^
Brown/Black	16 (28.6)	40 (71.4)		11	08–16	
Self-perceived school performance compared to peers						
Better than average	17 (43.6)	22 (56.4)	0.001 ^b^	13	09–16	<0.001 ^c^
Average/worse	6 (12.8)	41 (87.2)		09	06–12	
Income sufficiency						
Very well	3 (15.8)	16 (84.2)	0.084 ^b^	09	06–11	0.023 ^e^
Suitable	19 (34.5)	36 (65.5)		12	08–16	
Not well	1 (8.3)	11 (91.7)		08	07–12	
Receiving “Bolsa Familia” ^d^						
Yes	10 (17.9)	46 (82.1)	0.008 ^b^	10	07–13	0.009 ^c^
No	13 (44.8)	16 (55.2)		14	08–17	
Number of prenatal consultations ^d^						
≥6	7 (21.2)	26 (78.8)	0.334 ^b^	11	8–13	0.993 ^c^
<6	16 (30.8)	36 (69.2)		10	7–16	
Social Support						
Friends						
High	17 (31.5)	37 (68.5)	0.197 ^b^	12	08–16	0.200 ^c^
Low/none	6 (18.8)	26 (81.3)		09	07–14	
Grandparents						
High	13 (22.8)	44 (77.2)	0.247 ^b^	10	08–13	0.453 ^c^
Low/none	10 (34.5)	19 (65.5)		12	07–16	
Parents						
High	21 (33.9)	41 (66.1)	0.016 ^b^	12	08–16	0.052 ^c^
Low/none	2 (8.3)	22 (91.7)		09	07–11	
Partner						
High	14 (23.7)	45 (76.3)	0.350 ^b^	10	07–14	0.265 ^c^
Low/none	9 (33.3)	18 (66.7)		11	08–15	
Siblings						
High	18 (29.5)	43 (70.5)	0.366 ^b^	11	08–15	0.399 ^c^
Low/none	5 (20.0)	20 (80.0)		09	07–13	
Total	23 (26.7)	63 (73.3)		10.5	7.8–15.0	

^a^ SAHLPA score varies from 0–18, adequate health literacy was defined if the score was >14; ^b^ Chi-square test; ^c^ Mann–Whitney test; ^d^ One missing data; ^e^ Kruskal–Wallis test with post hoc Dunn’s test (suitable > very well, suitable > not well).

**Table 3:** Linear regression model for the associations among independent variables and the SAHLPA-18 score.

Variables	β	95%CI ^a^	*p* Value
Age categories			
Adolescents	−3.484	−5.006 to −1.962	<0.001
Adults	0		
Self-perceived school performance compared to peers			
Better than average	2.843	1.312 to 4.375	<0.001
Average/worse	0		
Income sufficiency			
Very well	0.498	−2.137 to 3.132	0.708
Suitable	2.775	0.568 to 4.983	0.014
Not well	0		

^a^ CI, confidence interval; SAHLPA-18, Short Assessment of Health Literacy for Portuguese-Speaking Adults (18 questions); Note, higher scores indicate higher level of health literacy.

## Appendix A

**Table A1:** Comparison of SAHLPA scores among adolescents and adults for each category of the independent variables (N = 86).

Characteristics	SAHLPA-18 Score (Range from 0–18)		
	Median (q_25–_q_75_)		
	Adolescents N = 41	Adults N = 45	*p* Value ^a^
Race/color ^b^			
White	8 (6–13)	11.5 (8–15.3)	0.084
Mixed-race/Black	9 (6–11)	15.5 (10–17)	<0.001
Self-perceived school performance compared to peers			
Better than average	9 (7–12)	16 (12–17)	<0.001
Average/worse	8 (5–10.3)	10 (7.5–15)	0.028
Income sufficiency			
Very well	6 (3–9)	10 (9–15.3)	0.022
Suitable	9 (7–12.5)	15 (10.8–17)	0.001
Not well	7 (4.5–9.5)	13 (9–14.5)	0.036
Receiving “Bolsa Família” ^b^			
Yes	9 (6–11)	11 (8.5–15.5)	0.015
No	7.5 (6.5–11.3)	16 (13–17)	<0.001
Number of prenatal consultations ^b^			
≥6	9 (6.5–11)	12.5 (10.3–16)	0.005
<6	8 (6–11.8)	15 (8.3–17)	0.001
Social Support			
Friends			
High	8 (5.5–10.8)	14.5 (10–16)	<0.001
Low/none	9 (6–12)	10 (9–17)	0.113
Grandparents			
High	9 (7–11)	13 (8.8–16)	0.001
Low/none	6.5 (4.5–13.3)	14 (9–17)	0.003
Parents			
High	8 (5–11.5)	15 (10.5–16)	<0.001
Low/none	8.5 (7–11)	9 (7.3–12.8)	0.787
Partner			
High	8 (5–11)	13 (9–16)	<0.001
Low/none	9 (7–13)	15 (10.3–17)	0.014
Siblings			
High	8 (5–11.3)	13 (10–16)	<0.001
Low/none	9 (6–11)	13.5 (7.8–16.3)	0.062

^a^ Mann–Whitney test; ^b^ One missing value.
